# Genome sequence of the phage-gene rich marine *Phaeobacter arcticus* type strain DSM 23566^T^

**DOI:** 10.4056/sigs.383362

**Published:** 2013-07-30

**Authors:** Heike M. Freese, Hajnalka Dalingault, Jörn Petersen, Silke Pradella, Karen Davenport, Hazuki Teshima, Amy Chen, Amrita Pati, Natalia Ivanova, Lynne A. Goodwin, Patrick Chain, John C. Detter, Manfred Rohde, Sabine Gronow, Nikos C. Kyrpides, Tanja Woyke, Thorsten Brinkhoff, Markus Göker, Jörg Overmann, Hans-Peter Klenk

**Affiliations:** 1 Leibniz Institute DSMZ – German Collection of Microorganisms and Cell Cultures, Braunschweig, Germany; 2 Los Alamos National Laboratory, Bioscience Division, Los Alamos, New Mexico, USA; 3 Biological Data Management and Technology Center, Lawrence Berkeley National Laboratory, Berkeley, California, USA; 4 DOE Joint Genome Institute, Walnut Creek, California, USA; 5 HZI – Helmholtz Centre for Infection Research, Braunschweig, Germany; 6 Institute for Chemistry and Biology of the Marine Environment, Oldenburg, Germany

**Keywords:** aerobic, psychrophilic, motile, high-quality draft, prophage-like structures, extrachromosomal elements, assimilatory nitrate reduction, *Alphaproteobacteria*, *Roseobacter* clade

## Abstract

*Phaeobacter arcticus* Zhang *et al*. 2008 belongs to the marine *Roseobacter* clade whose members are phylogenetically and physiologically diverse. In contrast to the type species of this genus, *Phaeobacter gallaeciensis*, which is well characterized, relatively little is known about the characteristics of *P. arcticus*. Here, we describe the features of this organism including the annotated high-quality draft genome sequence and highlight some particular traits. The 5,049,232 bp long genome with its 4,828 protein-coding and 81 RNA genes consists of one chromosome and five extrachromosomal elements. Prophage sequences identified *via* PHAST constitute nearly 5% of the bacterial chromosome and included a potential Mu-like phage as well as a gene-transfer agent (GTA). In addition, the genome of strain DSM 23566^T^ encodes all of the genes necessary for assimilatory nitrate reduction. Phylogenetic analysis and intergenomic distances indicate that the classification of the species might need to be reconsidered.

## Introduction

Strain 20188^T^ (DSM 23566^T^ = CGMCC 1.6500^T^ = JCM 14644^T^) is the type strain of *Phaeobacter arcticus*, a marine member of the *Rhodobacteraceae* (*Rhodobacterales*, *Alphaproteobacteria*) [1] which belongs to the *Roseobacter* clade, a phylogenetically and physiologically diverse group. Strain 20188^T^ was isolated from marine sediment of the Arctic Ocean (at 75° 00' 24'' N and 169° 59' 37'' W) from a water depth of 167 m. The species epithet is derived from the Latin adjective *arcticus* (= northern, arctic), referring to the site from where the strain was isolated. PubMed records do not indicate any follow-up research with strain 20188^T^ after its initial description and the valid publication of the new species name *P. arcticus* [1]. A few additional strains have been isolated and 16S rRNA gene sequenced (NCBI database), but no additional information on these strains is available so far. As a consequence, little is known regarding the physiology or distinguishing characteristics of *P. arcticus*. Here we present a summary classification and a set of features for *P. arcticus* DSM 23566^T^, together with the description of the high-quality permanent draft genome sequence and annotation, including insights into extrachromosomal elements, prophage-like structures as well as evidence for inorganic nitrogen assimilation.

## Classification and features

### 16S rRNA analysis

A representative genomic 16S rRNA gene sequence of *P. arcticus* DSM 23566^T^ was compared using NCBI BLAST [2,3] under default settings (e.g., considering only the high-scoring segment pairs (HSPs) from the best 250 hits) with the most recent release of the Greengenes database [4]. The relative frequencies of taxa and keywords (reduced to their stem [5]) were determined, weighted by BLAST scores. The most frequently occurring genera were *Phaeobacter* (46.4%), *Roseobacter* (24.9%), *Ruegeria* (6.1%), *Paracoccus* (5.4%) and *Leisingera* (4.4%) (91 hits in total). Regarding the nine hits to sequences from other members of the genus, the average identity within HSPs was 97.1%, whereas the average coverage by HSPs was 99.5%. Among all other species, the one yielding the highest score was 'marine bacterium ATAM407_56' isolated from a culture of *Alexandrium tamarense* AF359535, which corresponded to an identity of 99.4% and an HSP coverage of 99.9% (Note that the Greengenes database uses the INSDC (= EMBL/NCBI/DDBJ) annotation, which is not an authoritative source for nomenclature or classification). The highest-scoring environmental sequence was EU287348 (Greengenes short name 'Pacific arctic surface sediment clone S26-48'), which showed an identity of 99.9% and an HSP coverage of 100.0%. The most frequently occurring keywords within the labels of all environmental samples which yielded hits were 'marin' (5.6%), 'water' (5.5%), 'microbi' (4.5%), 'ocean' (4.5%) and 'coastal' (4.1%) (156 hits in total). The most frequently occurring keywords within the labels of those environmental samples which yielded hits of a higher score than the highest scoring species was 'arctic, pacif, sediment, surfac' (25.0%) (1 hit in total). These hits correspond to the known ecology of *P. arcticus* 20188^T^, which was isolated from marine sediment of the Arctic Ocean.

**Figure 1:**
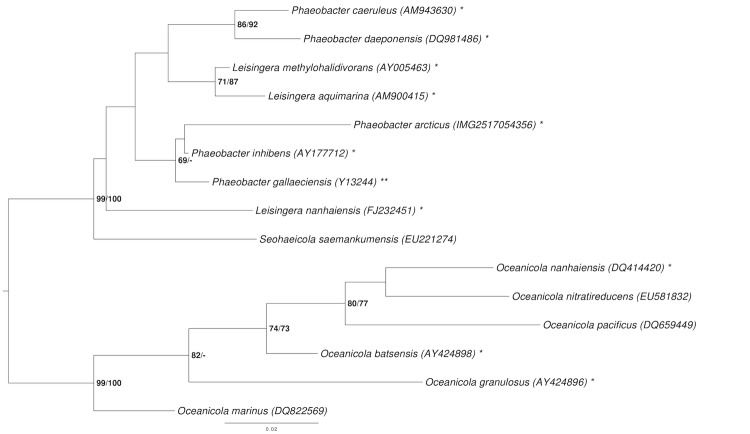
Phylogenetic tree highlighting the position of *P. arcticus* relative to the type strains of the other species within the genus *Phaeobacter* and neighboring genera such as *Leisingera*. The tree was inferred from 1,385 aligned characters [6,7] of the 16S rRNA gene sequence under the maximum likelihood (ML) criterion [8]. *Oceanicola* species were included in the dataset as outgroup taxa. The branches are scaled in terms of the expected number of substitutions per site. Numbers adjacent to the branches are support values from 1,000 ML bootstrap replicates [9] (left) and from 1,000 maximum-parsimony bootstrap replicates [10] (right) if larger than 60%. Lineages with type-strain genome sequencing projects registered in GOLD [11] are labeled with one asterisk, those also listed as 'Complete and Published' with two asterisks [12]. Two novel genome sequences were published in this issue [58,59].

The phylogenetic neighborhood of *P. arcticus* is shown in Figure 1 in a 16S rRNA gene tree. The sequences of the five 16S rRNA gene copies in the genome do not differ from each other, and differ by one nucleotide from the previously published 16S rDNA sequence DQ514304.

### Morphology and physiology

**Figure 2:**
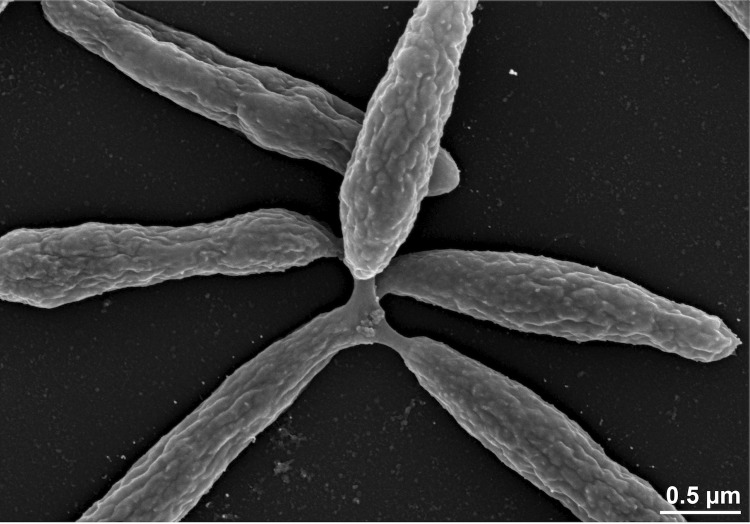
Scanning electron micrograph of *P. arcticus* DSM 23566^T^

The cells of strain 20188^T^ are motile rods with a width of 0.3 to 0.5 µm and a length of 1.0 to 2.6 µm (Figure 2, Table 1, [1]). Star-shaped cell aggregates occur (Figure 2). Colonies are circular and yellow. Growth occurs under psychrophilic, chemoheterotrophic and aerobic conditions and between 0°C and 25°C with an optimum growth rate at 19-20°C. No growth is observed at temperatures above 37°C [1]. Optimal pH for growth is approximately pH 6.0–9.0 (total range pH 5.0-10.0), and growth occurs within a salinity range of 2% to 9% NaCl, but not in the absence of NaCl [1]. Several carbohydrates like glucose, glycerol, fructose, melezitose, L-arabinose, D-mannose, mannitol, gluconate, N-acetylglucosamine and malate are utilized as sole carbon source, whereas sucrose, lactose, galactose, trehalose and cellobiose but also leucine, serine and L-glutamate cannot be utilized as sole carbon sources [1]. Strain 20188^T^ produces acid from glucose and glycerol. Further metabolic traits are listed elsewhere [1].

**Table 1:** Classification and general features of *P. arcticus* DSM 23566^T^ according to the MIGS recommendations [13].

**MIGS ID**	Property	**Term**	**Evidence code**
		Domain *Bacteria*	TAS [14]
		Phylum *Proteobacteria*	TAS [15]
		Class *Alphaproteobacteria*	TAS [16,17]
	Current classification	Order *Rhodobacterales*	TAS [16,18]
		Family *Rhodobacteraceae*	TAS [16,19]
		Genus *Phaeobacter*	TAS [20,21]
	Species	Species *Phaeobacter arcticus*	TAS [1]
MIGS-12	Reference for biomaterial	Zhang et al. 2008	TAS [1]
MIGS-7	Subspecific genetic lineage (strain)	20188^T^	TAS [1]
	Gram stain	Gram-negative	TAS [1]
	Cell shape	rod-shaped	TAS [1]
	Motility	motile	TAS [1]
	Sporulation	not reported	
	Temperature range	0-25°C, psychrophile	TAS [1]
	Optimum temperature	19-20°C	TAS [1]
	Salinity	2-9% (w/v) NaCl	TAS [1]
MIGS-22	Relationship to oxygen	aerobe	TAS [1]
	Carbon source	glucose; glycerol, mannitol, gluconate, malate	TAS [1]
	Energy metabolism	chemoheterotrophic	TAS [1]
MIGS-6	Habitat	marine sediment	TAS [1]
MIGS-6.2	pH	5.0-10.0, optimum 6.0-9.0	TAS [1]
MIGS-15	Biotic relationship	not reported	
MIGS-14	Known pathogenicity	none	IDA
MIGS-16	Specific host	not reported	
MIGS-18	Health status of host	not reported	
	Biosafety level	1	TAS [22]
MIGS-19	Trophic level	heterotroph	TAS [1]
MIGS-23.1	Isolation	marine sediment	TAS [1]
MIGS-4	Geographic location	Arctic Ocean	TAS [1]
MIGS-5	Time of sample collection	August 2003	TAS [1]
MIGS-4.1	Latitude	75.01	TAS [1]
MIGS-4.2	Longitude	-169.99	TAS [1]
MIGS-4.3	Depth	167 m	TAS [1]
MIGS-4.4	Altitude	167 m	NAS

Evidence codes - IDA: Inferred from Direct Assay; TAS: Traceable Author Statement (i.e., a direct report exists in the literature); NAS: Non-traceable Author Statement (i.e., not directly observed for the living, isolated sample, but based on a generally accepted property for the species, or anecdotal evidence). Evidence codes are from the Gene Ontology project [60].

### Chemotaxonomy

Ubiquinone-10 was found as major respiratory quinone, which is a common feature in most *Alphaproteobacteria*. The spectrum of main polar lipids in strain 20188^T^ consisted of phosphatidylethanolamine, phosphatidylglycerol, phosphatidylcholine and an unidentified aminolipid [1]. The major fatty acids are the monounsaturated fatty acids C_18:1 ω7c_ (44.63%) and 11-methyl C_18:1 ω7c_ (18.10%), followed by an unknown fatty acid (equivalent chain-length (ECL) of 11.799; 10.88%), C_16:0_ (9.69%), some hydroxyl fatty acids C_10:0 3-OH_ (6.75%), C_16:0 2-OH_ (3.95%), iso-C_15:0 2-OH_ and/or C_16:1ω7c_ (2.30%), as well as traces of C_15:0_, C_12:0_, C_18:1 2-OH_ and C_18:0_ [1]. The presence of photosynthetic pigments has not been tested.

## Genome sequencing and annotation

### Genome project history

This organism was selected for sequencing on the basis of the DOE Joint Genome Institute Community Sequencing Program 2010, CSP 441: “Whole genome type strain sequences of the genera *Phaeobacter* and *Leisingera* – a monophyletic group of physiologically highly diverse organisms”. The genome project is deposited in the Genomes On Line Database [11] and the complete genome sequence is deposited in GenBank. Sequencing, finishing and annotation were performed by the DOE Joint Genome Institute (JGI). A summary of the project information is shown in Table 2.

**Table 2:** Genome sequencing project information

MIGS ID	Property	Term
MIGS-31	Finishing quality	permanent draft
MIGS-28	Libraries used	One Illumina Standard (short PE) library, one Illumina CLIP (long PE) library
MIGS-29	Sequencing platforms	Illumina GAii, PacBio
MIGS-31.2	Sequencing coverage	Illumina 739 ×
MIGS-30	Assemblers	Allpaths version r39750, Velvet 1.1.05, phrap version SPS - 4.24
MIGS-32	Gene calling method	Prodigal 1.4, GenePRIMP
	INSDC ID	pending
	GenBank Date of Release	pending
	GOLD ID	Gi10722
	NCBI project ID	81437
	Database: IMG	2516653081
MIGS-13	Source material identifier	DSM 23566
	Project relevance	Tree of Life, carbon cycle, sulfur cycle, environmental

### Growth conditions and DNA extractions

A culture of DSM 23566^T^ was grown in DSMZ medium 514 (Bacto Marine Broth) [23] at 20°C. gDNA was purified using Jetflex Genomic DNA Purification Kit (GENOMED 600100) following the directions provided by the supplier but modified by the addition of 20 µl Proteinase K for cell lysis. The purity, quality and size of the bulk gDNA preparation were assessed by JGI according to DOE-JGI guidelines. DNA is available through the DNA Bank Network [24].

### Genome sequencing and assembly

The draft genome sequence was generated using Illumina data [25]. For this genome, we constructed and sequenced an Illumina short-insert paired-end library with an average insert size of 247 ± 59 bp which generated 16,028,960 reads and an Illumina long-insert paired-end library with an average insert size of 8,186 ± 3,263 bp which generated 9,112,084 reads totaling 3,771 Mbp of data (Feng Chen, unpublished). All general aspects of library construction and sequencing can be found at the JGI web site [26]. The initial draft assembly contained 20 contigs in 12 scaffolds. The initial draft data were assembled with Allpaths [27], version 39750, and the consensus was computationally shredded into 10 Kbp overlapping fake reads (shreds). The Illumina draft data were also assembled with Velvet [28], and the consensus sequences were computationally shredded into 1.5 Kbp overlapping fake reads (shreds). The Illumina draft data were assembled again with Velvet using the shreds from the first Velvet assembly to guide the next assembly. The consensus from the second Velvet assembly was shredded into 1.5 Kbp overlapping fake reads. The fake reads from the Allpaths assembly and both Velvet assemblies and a subset of the Illumina CLIP paired-end reads were assembled using parallel phrap (High Performance Software, LLC). Possible mis-assemblies were corrected with manual editing in Consed [29-31]. Gap closure was accomplished using repeat resolution software (Wei Gu, unpublished), and sequencing of bridging PCR fragments with Sanger and/or PacBio (Cliff Han, unpublished) technologies. A total of 13 PCR PacBio consensus sequences were completed to close gaps and to raise the quality of the final sequence. The final assembly is based on 3,771 Mbp of Illumina draft data, which provides an average 739× coverage of the genome.

### Genome annotation

Genes were identified using Prodigal [32] as part of the JGI genome annotation pipeline [33], followed by a round of manual curation using the JGI GenePRIMP pipeline [34]. The predicted CDSs were translated and used to search the National Center for Biotechnology Information (NCBI) nonredundant database, UniProt, TIGR-Fam, Pfam, PRIAM, KEGG, COG, and InterPro databases. Additional gene prediction analysis and functional annotation was performed within the Integrated Microbial Genomes - Expert Review (IMG-ER) platform [35].

## Genome properties

The genome statistics are provided in Table 3 and Figure 3. The genome consists of a 4,215,469 bp long chromosome (cArct_4215) and five extrachromosomal elements with 279,891 bp, 228,923 bp, 203,324 bp, 92,209 bp and 29,416bp length, respectively (pArct_A280 - pArct_E29), with a G+C content of 59.3% (Table 3 and Figure 3). The identification of the scaffolds as chromosome and as extrachromosomal elements is explained below. Of the 4,909 genes predicted, 4,828 were protein-coding genes, and 81 RNAs; 102 pseudogenes were also identified. Although the five 16S rRNA gene copies in the genome were identical, one of the adjacent 16S-23S rRNA gene internal transcribed spacer (ITS) differs in five nucleotides from the four other copies. The majority of the protein-coding genes (77.7%) were assigned a putative function while the remaining ones were annotated as hypothetical proteins. The distribution of genes into COGs functional categories is presented in Table 4.

**Table 3:** Genome Statistics

**Attribute**	**Value**	**% of Total**
Genome size (bp)	5,049,232	100.00
DNA coding region (bp)	4,429,124	87.72
DNA G+C content (bp)	2,992,500	59.27
Number of replicons	6	
Extrachromosomal elements	5	
Total genes	4,909	100.00
RNA genes	81	1.65
rRNA operons	5	
tRNA genes	59	1.20
Protein-coding genes	4,828	98.35
Pseudo genes	102	2.08
Genes with function prediction	3,814	77.69
Genes in paralog clusters	1,947	39.66
Genes assigned to COGs	3,755	76.49
Genes assigned Pfam domains	4,009	81.67
Genes with signal peptides	1,651	33.63
Genes with transmembrane helices	1,024	20.86
CRISPR repeats	0	

**Figure 3a:**
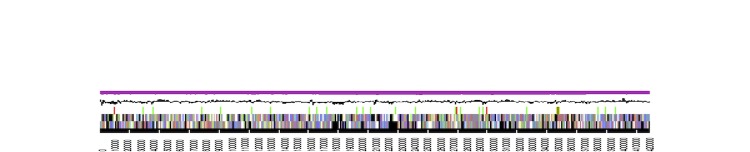
Graphical map of the *Phaeobacter arcticus* DSM 23566^T^ chromosome cArct_4215. From bottom to the top: Genes on forward strand (color by COG categories), Genes on reverse strand (color by COG categories), RNA genes (tRNAs green, rRNAs red, other RNAs black), GC content, GC skew.

**Table 4:** Number of genes associated with the general COG functional categories

**Code**	**Value**	**%age**	**Description**
J	180	4.36	Translation, ribosomal structure and biogenesis
A	0	0	RNA processing and modification
K	326	7.89	Transcription
L	186	4.50	Replication, recombination and repair
B	2	0.05	Chromatin structure and dynamics
D	37	0.90	Cell cycle control, cell division, chromosome partitioning
Y	0	0	Nuclear structure
V	52	1.26	Defense mechanisms
T	161	3.90	Signal transduction mechanisms
M	207	5.01	Cell wall/membrane/envelope biogenesis
N	54	1.31	Cell motility
Z	1	0.02	Cytoskeleton
W	0	0	Extracellular structures
U	90	2.18	Intracellular trafficking, secretion, and vesicular transport
O	160	3.87	Posttranslational modification, protein turnover, chaperones
C	265	6.41	Energy production and conversion
G	180	4.36	Carbohydrate transport and metabolism
E	452	10.94	Amino acid transport and metabolism
F	82	1.98	Nucleotide transport and metabolism
H	177	4.28	Coenzyme transport and metabolism
I	292	7.07	Lipid transport and metabolism
P	186	4.50	Inorganic ion transport and metabolism
Q	161	3.90	Secondary metabolites biosynthesis, transport and catabolism
R	514	12.44	General function prediction only
S	367	8.88	Function unknown
-	1,154	23.51	Not in COGs

**Figure 3b:**
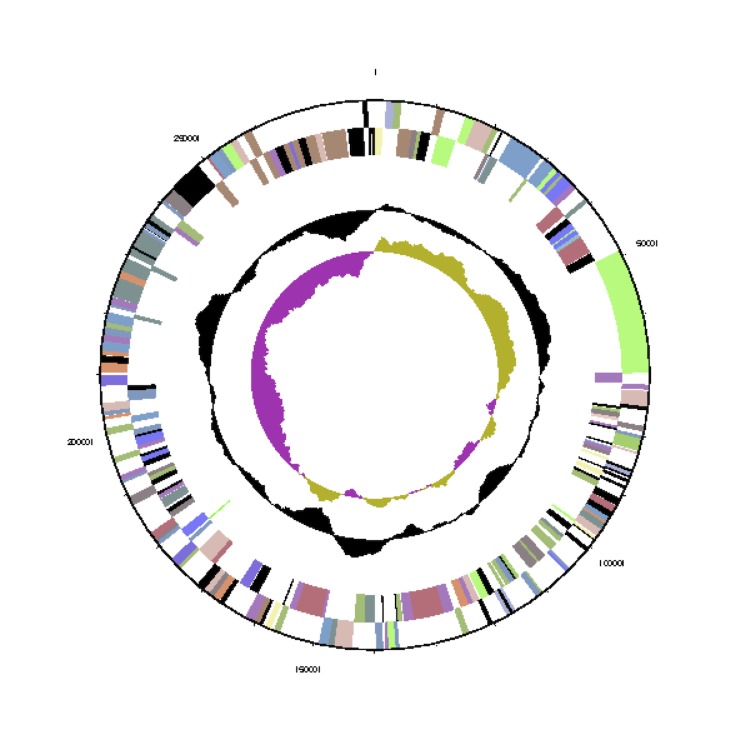
Graphical map of the *Phaeobacter arcticus* DSM 23566^T^ extrachromosomal element pArct_A280. From outside to the center: Genes on forward strand (color by COG categories), Genes on reverse strand (color by COG categories), RNA genes (tRNAs green, rRNAs red, other RNAs black), GC content, GC skew.

**Figure 3c:**
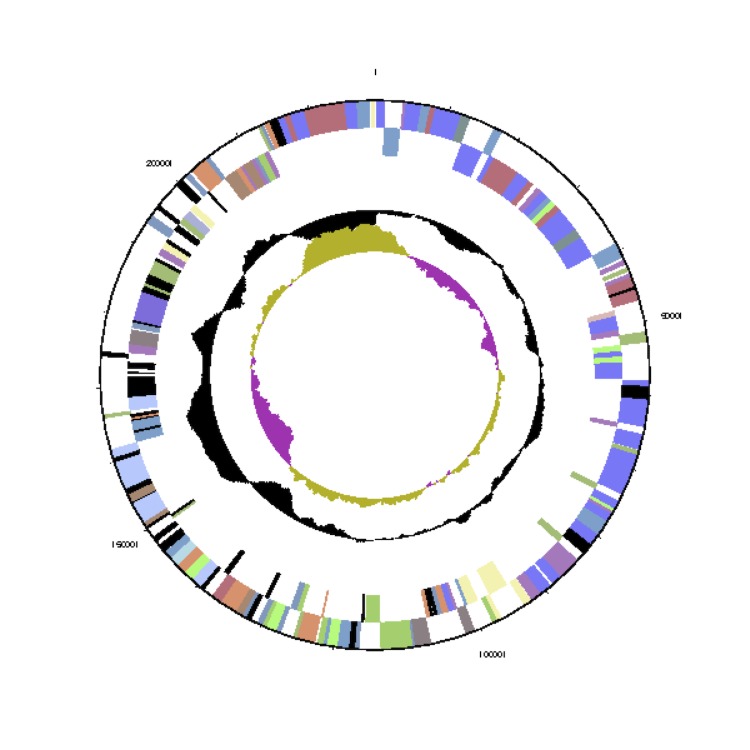
Graphical map of the *Phaeobacter arcticus* DSM 23566^T^ extrachromosomal element pArct_B229. From outside to the center: Genes on forward strand (color by COG categories), Genes on reverse strand (color by COG categories), RNA genes (tRNAs green, rRNAs red, other RNAs black), GC content, GC skew.

**Figure 3d:**
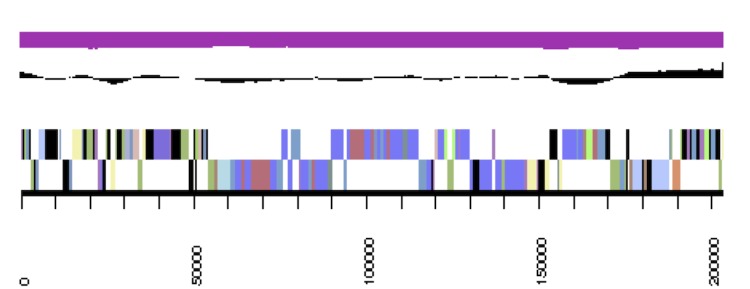
Graphical map of the *Phaeobacter arcticus* DSM 23566^T^ extrachromosomal element pArct_C203. From bottom to the top: Genes on forward strand (color by COG categories), Genes on reverse strand (color by COG categories), RNA genes (tRNAs green, rRNAs red, other RNAs black), GC content, GC skew.

**Figure 3e:**
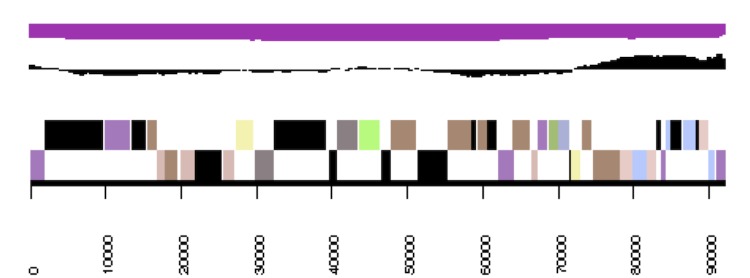
Graphical map of the *Phaeobacter arcticus* DSM 23566^T^ extrachromosomal element pArct_D92. From bottom to the top: Genes on forward strand (color by COG categories), Genes on reverse strand (color by COG categories), RNA genes (tRNAs green, rRNAs red, other RNAs black), GC content, GC skew.

**Figure 3f:**
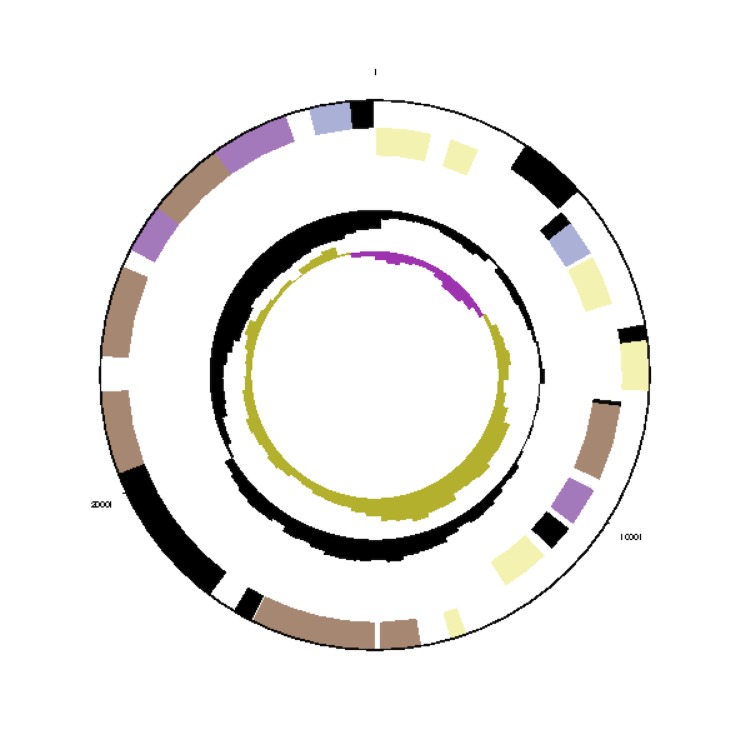
Graphical map of the *Phaeobacter arcticus* DSM 23566^T^ extrachromosomal element pArct_E29. From outside to the center: Genes on forward strand (color by COG categories), Genes on reverse strand (color by COG categories), RNA genes (tRNAs green, rRNAs red, other RNAs black), GC content, GC skew.

## Insights into the genome

The replication-initiation systems identified on the scaffolds were as follows: cArct_4215, dnaA; pArct_A280, repB-I pArct_B229, repABC-5; pArct_C203, repABC-9; pArct_D92, repA-I; pArct_E29, repA-III, repA-IV and repB-III. This justifies the interpretation of cArct_4215 as (potentially circular) chromosome and of the other scaffolds as (potentially circular) extrachromosomal elements [36,37]. 

### Nitrogen metabolism

Although it was reported that strain 20188^T^ did not reduce nitrate [1], the enzymes required for nitrate reduction and metabolism of other nitrogen oxides are encoded in the genome of DSM 23566^T^. The presence of nitrate reductase (*narGHIJ*, Phaar_00816 - Phaar_00819; *nasA*, Phaar_03836) and nitrite reductase (NAD(P)H) (*nirBD*; Phaar_03837, Phaar_03838) suggests the capacity for assimilatory nitrate reduction, i.e. reduction of nitrate *via* nitrite to ammonium [38]. Interestingly, only a copper-type nitrite reductase gene, analogous to *nirK* in *P. gallaeciensis* [39], is missing to complete the pathway for potential denitrification from nitrate to nitrogen. In addition to the above mentioned nitrate reductase genes, nitric oxide reductase (*norBCDQ*; Phaar_00646 - Phaar_00649) and, in contrast to *P. gallaeciensis*, even nitrous oxide reductase genes (*nosDZ*; Phaar_02837, Phaar_02838) are present, indicating the potential to reduce nitric oxide *via* nitrous oxide to nitrogen [40].

Small methylated amines are also considered as potential nitrogen source for many members of the marine *Roseobacter* clade [41]. In contrast to *L. nanhaiensis* DSM 24252^T^ (IMG object ID 2521172577), no methylamine-utilizing genes could be detected in *P. arcticus* strain DSM 23566^T^, nor in *P. gallaeciensis*. When using the suggested protein sequences for trimethylamine monooxygenase (Tmm, ACK52489) and GMA synthetase (GmaS, BAF99006) [41] as query in the BLAST in the IMG database [42,43] no hits (≥e^-80^ [44],) were found. Lower e-value cutoffs (> e-30) yielded some hits but in contrast to methylamine-utilizing genes [41], these hits were not clustered together.

Although the strain did not grow with serine, L-glutamate or leucine as single substrate [1], L-serine dehydratase (EC:4.3.1.17, Phaar_02408) and threonine dehydratase (EC:4.3.1.19, Phaar_00247, _03532, _03664) genes, which catalyze the conversion of serine to pyruvate are found. The glutamate dehydrogenase (NAD(P)+) (EC:1.4.1.3, Phaar_00693) gene degrading L-glutamate to 2-oxoglutarate is also present in the genome sequence. However, we cannot exclude a putative lack of respective transport systems. For leucine degradation, all but one gene is present; dihydrolipoamide transacylase (EC:2.3.1.168). When using the respective protein sequence from the leucine utilizer *Paracoccus denitrificans* PD1222 as query through BLASTP, no hits were found in strain DSM 23566^T^. Interestingly, in *P. daeponensis* (IMG object ID 2521172619) which is known to grow with leucine, but also in *P. caeruleus* (IMG object ID 2512047087) the respective gene is located on an extrachromosomal element by which all genes of the leucine degradation pathway are found. 

### Mobile genetic elements

Genomic diversification of bacteria is known to be driven by phage-mediated horizontal gene transfer. Prophage-like structures are found in many (marine) bacteria [45,46]. In strain DSM 23566^T^, 58 genes were annotated as phage genes. This number is distinctly higher than those in the phylogenetically related *Phaeobacter* and *Leisingera* species (Figure 1; 8 – 38 phage genes) and in other *Roseobacter* clade bacteria [47]. Analysis of the genome of strain DSM 23566^T^ with PHAST [48] revealed eight prophage regions, two of which were intact, another four of which were questionable and two that were incomplete (Table 5). These prophage regions constituted nearly 5% of the bacterial chromosome (cArct_4215). One of the intact prophage regions (7) is likely a Mu-like phage, since many of the coding sequences (mostly corresponding to Phaar_02143 - Phaar_02190) yielded hits with *Rhodobacter* phage RcapMu (NC_016165), *Enterobacteria* phage Mu (NC_000929) and *Burkholderia* phage BcepMu (NC_005882). The incomplete prophage region 3 also had hits to Mu-like phages. Mu-like phages are known to pack and transfer flanking host DNA in addition to their own genome and are found in *Rhodobacter capsulatus,* although they are more common in *Gammaproteobacteria* [49].

**Table 5:** Prophage regions in the genome of *P. arcticus* DSM 23566^T^ cArct_4215, GC% = 59.10%, length = 4,215,469 bp^†^

Region	Region-Length	Completeness	Score	#CDS	Region-Position	Specific Keyword	GC-%
1	14.7 Kb	questionable	70	18	3284-18065	fiber, tail, head, lysin	60.91%
2	22.0 Kb	incomplete	50	22	1599730-1621795	integrase, terminase	58.75%
3	18.5 Kb	incomplete	40	22	1804950-1823500	transposase	55.77%
4	17.0 Kb	intact	100	20	1905214-1922300	capsid, fiber, tail, head, Portal, terminase, protease	62.27%
5	33.8 Kb	questionable	90	37	2111516-2145342	integrase, tail, head, terminase, lysin	59.92%
6	31.3 Kb	questionable	70	25	2203367-2234719	integrase, tail, transposase	57.69%
7	33.3 Kb	intact	110	46	2247246-2280565	tail, plate, transposase, portal, terminase, protease	58.80%
8	33.5 Kb	questionable	90	19	2437800-2471330	integrase, fiber, tail, head, lysin	60.17%

† COMPLETENESS, a prediction of whether the region contains an intact or incomplete prophage based on the applied criteria of PHAST; SCORE, the score of the region based on the applied criteria of PHAST; #CDS, the number of coding sequence; REGION_POSITION, the start and end positions of the region on the bacterial chromosome; GC-%, the percentage of GC nucleotides of the region.

The other intact prophage region (region 4 in Table 5) strongly resembles a GTA (gene transfer agent) since it contains a major capsid protein (PhaarD_01806) that is similar (64%, e=0 [42,43]) to the highly conserved major capsid protein (g5) of *R. capsulatus* GTA [50,51]. These phage-like entities contain and transfer random fragments of bacterial host genomic DNA and are found in most *Alphaproteobacteria*, especially in the *Rhodobacterales* [50]. The occurrence of all these prophage-like structures together with the absence of a CRISPR system (i.e. an antiphage defense system [52]) suggests that phages may be important for genomic diversification within the *Phaeobacter* group.

### Secondary metabolism

In contrast to its relative *P. gallaeciensis,* which is known for the production of the antibiotic tropodithietic acid (TDA) [39], no homologs of TDA production genes *tdaBCEF* were found in strain DSM 23566^T^. However, Phaar_00595 shared homology (e<10^-80^) with a lantibiotic biosynthesis protein LanM, and four genes (Phaar_00296, _00590, _01696, _01697) were homologous to bacteriocin/lantibiotic exporters indicating the production of peptide antibiotics [53,54].

### Classification

As the 16S rRNA gene analysis (Figure 1) indicated intermixed positions of *Phaeobacter* and *Leisingera* species (even though with low bootstrap support), the classification of the group might need to be reconsidered. We thus conducted a preliminary phylogenomic analysis using GGDC [55-57] and the draft genomes of the type strains of the other *Leisingera* and *Phaeobacter* species. The results shown in Table 6 indicate that the DNA-DNA hybridization (DDH) similarities calculated *in silico* of *P. articus* to other *Phaeobacter* species are, on average, not higher than those to *Leisingera* species. The highest value is actually obtained for *L. nanhaiensis* and formula 2, which is preferred if genomes are only incompletely sequenced [55]. The overall low similarity values indicate that *P. arcticus* might better be placed in a separate genus, particularly if compared to the according similarity values between the other *Leisingera* and *Phaeobacter* species [58,59].

**Table 6:** DDH similarities between *P. arcticus* DSM 23566^T^ and the other *Phaeobacter* and *Leisingera* species (with genome-sequenced type strains) calculated *in silico* with the GGDC server version 2.0 [55].

**Reference species**	**formula 1**	**formula 2**	**formula 3**
*L. aquamarina* (2521172617)	16.60±3.25	20.70±2.32	16.50±2.75
*L. methylohalidivorans* (2512564009)	17.20±3.28	20.40±2.32	17.00±2.77
*L. nanhaiensis* (2521172577)	14.60±3.12	22.90±2.37	14.80±2.66
*P. caeruleus* (2512047087)	16.90±3.26	20.40±2.32	16.70±2.76
*P. daeponensis* (2521172619)	17.00±3.27	21.00±2.33	16.90±2.77
*P. gallaeciensis* (AOQA01000000)	16.40±3.24	21.80±2.35	16.40±2.75
*P. inhibens* (2516653078)	16.20±3.22	20.80±2.33	16.10±2.73

The standard deviations indicate the inherent uncertainty in estimating DDH values from intergenomic distances based on models derived from empirical test data sets (which are always limited in size); see [57] for details. The distance formulas are explained in [55]. The numbers in parentheses are IMG object IDs (GenBank accession number in the case of *P. gallaeciensis*) identifying the underlying genome sequences.
